# Cyclooxygenase-mediated IL-10Rα signaling underlies pro-tumor phenotypes of macrophages in glioma

**DOI:** 10.1038/s41419-026-09072-7

**Published:** 2026-07-27

**Authors:** Jiahui Zhou, Bingxi Shi, Qiangsheng Li, Fangzhen Li, Zhiwen Zhang, Qiuhong Zhu, Huipeng Wang, Wei Wang, Hai-bo Wu, Aili Zhang, Bofeng Li, Wenchao Zhou

**Affiliations:** 1 Department of Pathology, The First Affiliated Hospital of USTC, Division of Life Sciences and Medicine, University of Science and Technology of China, Hefei, China; 2 Intelligent Pathology Institute, The First Affiliated Hospital of USTC, Division of Life Sciences and Medicine, University of Science and Technology of China, Hefei, China; 3 Department of Medical Oncology, the First Affiliated Hospital of USTC, Division of Life Sciences and Medicine, University of Science and Technology of China, Hefei, China; 4 Henan Key Laboratory of Genetic and Developmental Disorders, Institute of Children’s Health, Henan Academy of Innovations in Medical Sciences, Zhengzhou, China; 5 Zhengzhou Hospital of Beijing Children’s Hospital, Zhengzhou, China; 6 Department of Cell Biology, School of Life Science, Anhui Medical University, Hefei, China; 7 Research Center for Translational Medicine, The Second Affiliated Hospital of Anhui Medical University, Hefei, China; 8 School of Basic Medical Sciences, Anhui Medical University, Hefei, China

**Keywords:** CNS cancer, Tumour immunology

## Abstract

Tumor-associated macrophages (TAMs) in glioblastoma (GBM) often adopt tumor-supportive phenotypes, but the underlying signaling in TAMs is unclear. Interleukin-10 (IL-10) is frequently upregulated in tumors, but the understanding of IL-10 in the tumor immune microenvironment is controversial. This study aims to determine the roles of IL-10 receptor alpha (IL-10Rα) signaling in TAMs and explore the targetable mediators for GBM treatment. We found that growth of intracranial GBMs was delayed in engineered mice with myeloid-specific knock-out of IL-10Rα, which was accompanied by changes in TAM phenotypes. Meanwhile, IL-10 expression in GBMs, which was largely accredited to TAMs, were not affected by IL-10Rα deficiency. RNA-sequencing analysis and immunostaining revealed cyclooxygenases COX-1/2 as potential downstream effectors of IL-10Rα in TAMs. Pharmacological inhibition of COX-1/2 suppressed bone-marrow derived macrophages (BMDM) polarization upon IL-4/IL-10 treatment. Consistently, the COX inhibitor NS-398 mitigated intracranial GBM growth. In summary, we not only demonstrated the crucial roles of IL-10Rα but also discovered COX-1/2 as downstream targets for pro-tumor phenotypes of TAMs in GBMs. These findings provide new clues for future development of immunotherapies for GBM treatment.

## Introduction

Glioblastoma (GBM) is the most common and malignant primary brain tumor with a median patient survival of 14-16 months [1]. Tumor immunotherapy has emerged as a promising therapeutic strategy for malignant tumors, but its outcomes largely depend on the tumor immune microenvironment [2]. Tumor-associated macrophages (TAMs) are the predominant immune population in GBM, which often exhibit tumor-supportive phenotypes and contribute to tumor growth by promoting angiogenesis, tumor cell proliferation, resistance against treatments, and immune escape [3–5]. Several studies have addressed the tumor cell-derived factors that promote tumor-supportive phenotypes of TAMs. For example, glioma stem cells produce periostin and WISP1 to program TAMs polarization probably through exosomes as delivery vehicles [6, 7]. Meanwhile, membrane receptors on TAMs such as integrins and CSF-1R respond to those tumor cell-derived stimuli and drive the M2 polarization [6, 8]. Despite these insights, the TAM-intrinsic signal pathways underlying tumor-supportive phenotypes of TAMs is poorly investigated. The mechanistic studies have been technically challenged because of the difficulties to isolate and culture sufficient numbers of TAMs. Therefore, myeloid-specific knockout of the genes of interest in mouse may be a highly useful tool to look into the TAM-intrinsic pathways underlying their tumor-supportive phenotypes.

Among a long list of factors with the capacity to induce the tumor-supportive M2 phenotype of macrophages in vitro, IL-10 is especially interesting. IL-10 plays essential roles in almost all anti-inflammatory processes that prevent tissue damages from inflammation [9]. Elevated expression of IL-10 in multiple types of cells in tumor tissues has been reported [10, 11]. Consistently, it has been shown that interference with IL10 signaling could suppress tumor development [12]. IL-10 is able to promote tumor cell proliferation and inhibit anti-tumor activities of lymphoid and myeloid immune cells, in which Jak1, Tyk2, and STAT3 act as key mediators [13, 14]. Induction of M2 macrophages in vitro upon combined stimulation of IL-4, IL-10, and IL-13 activates the similar pathways [15]. However, signaling at the downstream of the IL-10 receptor (IL-10R) controlling TAM differentiation is poorly understood. Interestingly, whereas IL-10 demonstrates multifaceted pro-tumor activities, controversial discoveries have unraveled the anti-tumor immune regulatory roles of IL-10 [11, 16]. IL-10 treatment of melanoma-bearing mice enhanced expansion and effector function of terminally exhausted CD8^+^ tumor-infiltrating lymphocytes, which synergized with adoptive T cell transfer immunotherapy and eradicated established tumors [16]. Furthermore, bacteria-induced production of IL-10 from TAMs with high IL-10R expression triggered expansion and activation of exhausted tumor-resident CD8^+^ T cells and effectively eliminated tumors [11]. Therefore, the roles of IL-10/IL-10R signaling in TAMs and the underlying mechanisms are worth a revisit.

In this study, using mice with *lyz2* promoter-driven myeloid-specific knock-out of *Il10ra*, we studied TAM polarization in orthotopic GBM and searched for molecular mediators of IL10-Rα signaling. Disruption of IL10-Rα in myeloid cells resulted in retarded tumor growth and decreased expression of M2 markers on TAMs. However, IL-10 production from TAMs was not affected by IL10-Rα deficiency. RNA-sequencing of macrophages derived from wildtype and myeloid-specific *Il10ra* knock-out mice revealed cyclooxygenases COX-1/2 as potential effectors at the downstream of IL10-Rα, which was confirmed by qPCR and immunostaining analyses. Furthermore, pharmacological inhibition of COX-1/2 suppressed tumor-supportive phenotypes of bone marrow-derived macrophages in vitro. Last but not least, the COX inhibitor NS-398 mitigated intracranial GBM growth. These findings provide new clues for future development of immunotherapies for GBM treatment.

## Materials and methods

### Myeloid-specific IL-10Rα knockout in mice

The myeloid-specific *Il10ra* deletion on a C57BL/6 background was achieved as previously described through homologous recombination [17, 18]. The mouse strain was sent to the Jackson Laboratory and named as *IL-10Rα*^*fl/f*^ (B6(SJL)-*Il10ra*^*tm1.1Tlg*^/J (https://www.jax.org/strain/028146). Briefly, loxp were put on both flanks of the third exon of the *Il10ra* gene. The expression of the cre protein was under the control of the *lyz2* promoter and specifically occurred in mature myeloid cells. *Il10ra*^flox/flox^ mice were crossed with Lyz2-cre^+^ mice to generate F1 heterozygous offspring. These F1 heterozygotes were then intercrossed to produce F2 progeny with myeloid cell-specific deletion of *Il10ra*, designated as IL-10Rα^Lysm^. Genotyping was performed with tail tissues from mice aged 4 weeks using primers for *I10ra* (5’-CCTCCATACGAGAGGTCTTGG-3’ and 5’-AGCTAACTGCCCTGGGACTC-3’) and cre (5’-CCCAGAAATGCCAGATTACG-3’ and 5’-CTTGGGCTGCCAGAATTTCTC-3’).

### Collection of murine macrophages and microglia

Femur and brain were collected from mice aged 6–8 weeks that were anesthetized and sterilized with 75% alcohol before tissue harvest, and macrophages and microglia were obtained according to previous publications [19, 20]. Bone-marrow cells were flushed out from femur and induced to macrophages. Brain tissues were cut into small pieces and dissociated into single cells using the Neural Tissue Dissociation Kit (Miltenyi Biotec, 130-093-231). Cells were then subjected to magnetic-activated cell sorting (MACS) with anti-CD11b magnetic beads (Miltenyi Biotec, 130-093-634) to obtain microglia.

### Mouse intracranial GBM and drug treatment

Mouse GL261 GBM cells were kind gifts from Professor Jianghong Man (National Center of Biomedical Analysis, Beijing) that were authenticated by STR. Cells were routinely tested for mycoplasma contamination and confirmed to be negative. GL261 cells were transduced with luciferase through lentiviral infection before implantation into mouse brains. Wild-type C57BL/6 mice were purchased from Hangzhou Ziyuan Company. Mice were maintained at 21 ± 1 °C in 40–70% humidity under a 12/12-hour light/dark cycle at the animal facility of University of Science and Technology of China. Mice of either sex aged 6–8 weeks were used for orthotopic GBM implantation. For intracranial tumor formation, 5000 GL261 cells were engrafted into the cerebral cortex positioned 2 mm to the right of the bregma and 3.5 mm below the surface of the skull through stereotactic injection of 5 µl cell suspension. Bioluminescent imaging was used to monitor intracranial tumor growth in mice by intraperitoneal injection of D-luciferin (150 mg/kg, Goldbio, LUCK-1G) followed by signal capture with the Spectrum IVIS imaging system (PerkinElmer). At the end of animal studies, tumor-bearing mice with neurological signs such as seizure, impaired movement, tail tone, etc., were euthanized through carbon dioxide inhalation.

For NS-398 treatment, 15 days after GL261 implantation, the tumor-bearing mice with similar levels of bioluminescent signals were divided randomly into two groups and treated with control or NS-398 (10 mg/kg, 100 μl in volume) through intraperitoneal injection every day until the demonstration of neurological signs. NS-398 was in solvent composed of 10% DMSO, 40% PEG300, 5% Tween-80, and 45% physiological saline solution.

Sample size was predetermined not with specific method but based on standard practice in the field. 5–10 mice per group were used to ensure robustness. Only animals with accidental death (for example, due to infection or intracranial injection) were excluded from the data analysis. The investigators were not blinded to allocation during experiments and outcome assessment.

### Immunofluorescence staining

Immunofluorescent staining was performed as described before [21]. Mouse intracranial xenografts were fixed overnight in 4% PFA at 4 °C, stored in 30% sucrose solution overnight at 4°C, embedded in OCT at -20 °C, and cryosectioned at a thickness of 7 microns. For blocking and permeabilization, sections were incubated in a PBS solution containing 5% donkey serum plus 0.3% Triton X-100 for 1 hour at room temperature. Sections were then incubated with primary antibodies (1:200 dilution) in PBS containing 5% donkey serum and 0.3% Triton-100 overnight at 4 °C. After then, the sections were washed with 0.1% TWEEN-20 in PBS and incubated with secondary antibodies (1:1,000 dilution) plus Hochest (1:20,000 dilution) in PBS containing 5% donkey serum for 2 h at room temperature in dark. After wash with 0.1% Tween-20 in PBS, a coverslip was mount on sections, and the staining was subjected to microscopy. Specific antibodies against Arg1 (Cell Signaling Technology, 93668), Iba1 (abcam, ab5076), IL-10 (Santa Cruz, sc-365858 and sc-8348), COX-1 (Santa Cruz, sc-19998), COX-2 (Santa Cruz, sc-19999), MHC-II (Biolegend, 307665), p-STAT3 (Cell Signaling Technology, 9145), CD3ε (Santa Cruz, 99940), CD31 (Dako,M082301), Cleaved Caspase-3 (Cell Signaling Technology, 9664) and Ki67 (Abcam, ab15580) were used for the staining of GBM tumor sections as indicated. Analysis of immunofluorescence staining was preformed using Image J software.

### Differentiation and treatment of bone marrow-derived macrophages

Bone marrow cells were flushed out from mouse femurs and cultured in 1640 medium supplemented with 10% FBS (Lonsera, S711-001S) and 100 U/ml penicillin–streptomycin (Biological Industries, 03-031-1B). For macrophage differentiation, the bone marrow cells were stimulated with 20 ng/ml M-CSF (BBI, C600148) for 5 days. After then, the cells were either subjected to further treatment with M-CSF, 20 ng/ml IL-4 (BBI, C600050) and 20 ng/ml IL-10 (BBI, C600051) for 3 days to induce M2-like state, or kept in M-CSF for 3 days to obtain M0-like macrophages. For inhibition of COX-1/2, cells were treated with Loxoprofen (5 μg/ml), NS-398 (5 μg/ml), or Tolfenamic Acid (10 μM) dissolved in DMSO for 24 h. Conditional medium treatment of macrophages was performed according to previous publications with minor modification [22]. For collection of GL261 conditioned medium, 1 million cells were cultured in a 10 cm dish in 7 mL DMEM medium for 48 h, and the supernatant was collected and centrifuged at 4000 rpm for 10 min to remove cell debris. The resultant conditioned medium was then used to culture the abovementioned M0-like macrophages at a concentration of 8 × 10^5^ cells per well in 6-well plates at the presence or absence of the COX-1/2 inhibitors.

### Quantitative PCR

Total RNA was extracted using EZ-10 Spin Column Total RNA Isolation Kit (BBI, B610583-0100). Complementary DNA was synthesized using PrimeScript™ RT Master Mix (TAKARA, RR036A). qPCR was performed using SYBR Green qPCR Mix (Biosharp, BL705A) on a LightCycler 96 Real Time PCR instrument (Roche). Cycle threshold (Ct) values were determined automatically by the instrument. Relative gene expression was calculated by the 2^-ΔΔCt^ method with *Rpl13a* as the internal control. At least three biological repeats were performed for each analysis, whereas a representative result containing three technical replicates were used to generate graphs. The sequences of qPCR primers are as the following. *Mrc1* (CD206): 5’-CTCTGTTCAGCTATTGGACGC-3’; 5’-CGGAATTTCTGGGATTCAGCTTC-3’. *Rpl13a*: 5’-AGCCTACCAGAAAGTTTGCTTAC-3’; 5’-GCTTCTTCTTCCGATAGTGCATC-3’. *Il10*: 5’-GCTCTTACTGACTGGCATGAG-3’; 5’-CGCAGCTCTAGGAGCATGTG-3’. *Ptgs1* (Cox-1): 5’-ATGAGTCGAAGGAGTCTCTCG-3’; 5’-GCACGGATAGTAACAACAGGGA-3’. *Ptgs2* (Cox-2): 5’-TTCAACACACTCTATCACTGGC-3’; 5’-AGAAGCGTTTGCGGTACTCAT-3’. *Cd163*: 5’-GGTGGACACAGAATGGTTCTTC-3’; 5’-CCAGGAGCGTTAGTGACAGC-3’. *Cd80*: 5’-TGCTGCTGATTCGTCTTTCAC-3’; 5’-GAGGAGAGTTGTAACGGCAAG-3’. *Il6*: 5’-CCAGTTGCCTTCTTGGGACT-3’; 5’-GGTCTGTTGGGAGTGGTATCC-3’. *Tnfa*: 5’-ACGGCATGGATCTCAAAGAC-3’; 5’-GTGGGTGAGGAGCACGTAGT-3’. *Nos2* (iNOS): 5’-ACATCGACCCGTCCACAGTAT-3’; 5’-CAGAGGGGTAGGCTTGTCTC-3’.

### RNA-seq and differential expression analysis

Total RNA was extracted with TRIzol Reagent (BBI, B610409) according to the company’s instructions. RNA quality was confirmed through measurement with Agilent Bioanalyzer. Differential expression analysis was performed in R (version 4.2.2) using the DESeq2 package (version 3.13) with the design formula ~ batch + group. Differentially expressed genes (DEGs) were visualized using the ComplexHeatmap package. To search for the mediators at the downstream of IL10-Rα in macrophages, genes that showed log₂FC > 1 in M2-like compared to M0-like macrophages from wildtype mice and log₂FC > 0 in M2-like compared to M0-like macrophages from IL-10Rα^Lysm^ mice were picked up. The genes with attenuated upregulation in M2-like macrophages from IL-10Rα^Lysm^ relative to wildtype mice were further subjected to functional enrichment analysis. GO enrichment analysis for Biological Process (BP) terms was conducted using the enrichGO function in clusterProfiler (OrgDb = org.Mm.eg.db), and KEGG pathway analysis was performed using the enrichKEGG function (organism = “mmu”). Visualization was achieved with ggplot2 and ggrepel, and key genes such as *Ptgs1* and *Ptgs2* were highlighted in the volcano plots.

### Public database and bioinformatics analysis

Patient gene expression matrices and corresponding clinical survival information were downloaded from the Chinese Glioma Genome Atlas (CGGA) database (http://www.cgga.org.cn/) and analyzed using R (version 4.2.2). Kaplan–Meier survival models were constructed with the *survival* and *survminer* packages. The optimal cutoff point was automatically determined using the surv_cutpoint function, based on which patients were stratified into high- and low-expression groups, with statistical significance evaluated via the log-rank test.

To assess the combined prognostic impact of PTGS1/2 and the macrophage marker gene AIF1, patients were grouped using the surv_cutpoint function to compute the optimal cutoff based on expression levels. Patients were then classified into four subgroups according to the high and low expressions of PTGS1/2 and AIF1, followed by Kaplan–Meier survival analysis.

Correlation analysis between PTGS1/2 and IL10-RA was conducted using the Cancer Genome Atlas (TCGA)-GBM dataset [23]. Pearson correlation coefficients were calculated with the *ggstatsplot* package to evaluate linear correlations between genes. Scatter plots with regression lines were generated using the *ggpubr* package.

Single-cell RNA sequencing data of GBM patients were from public Gene Expression Omnibus (GEO) databases (GSE223065, GSE135045). Data was preprocessed with *Seurat* v4, and batch effects were removed with the *Harmony* algorithm. Dimensionality reduction and single-cell clustering were conducted using the RunUMAP function of the *Seurat* R package, with annotation of cell populations based on canonical marker genes. Visualizations were performed with ggplot2, whereas uniform manifold approximate projection (UMAP) coordinates and gene expression values were obtained using the FetchData function for afterward analysis and visualization.

Alternatively, scRNA-seq datasets from GEO database (GSE103224, GSE138794, GSE139448, GSE141385, GSE141982, and GSE148842) was analyzed and visualized using the TISCH2 online module. In brief, TISCH2 adopted a uniform analysis pipeline MAESTRO for quality control, clustering and cell-type annotation, followed by curation of the cell-type annotation of all datasets at the levels of malignancy, major-lineage and minor-lineage, which makes gene expression in different cell types comparable across all datasets. For analysis of gene expression, we used the Average Gene Expression mode with the setting of All Lineages, which performed normalization based on Transcripts Per Million (TPM). Average gene expressions in specific cell types were demonstrated as log(TPM/10 + 1).

### Statistics

The level of significance was determined by the unpaired two-tailed Student’s t test, or two-tailed log-rank test with α = 0.05 (Kaplan-Meier survival curves) with the GraphPad Prism 8 software. All quantitative data presented are the mean ± s.d. *P* < 0.05 is considered statistically significant. Precise experimental details (number of animals or cells, experimental replication, and *p* values) are provided in the figure legends.

## Results

### Myeloid knock-out of IL-10Rα inhibited GBM growth and affected phenotypes of TAMs

**Fig. 1:**
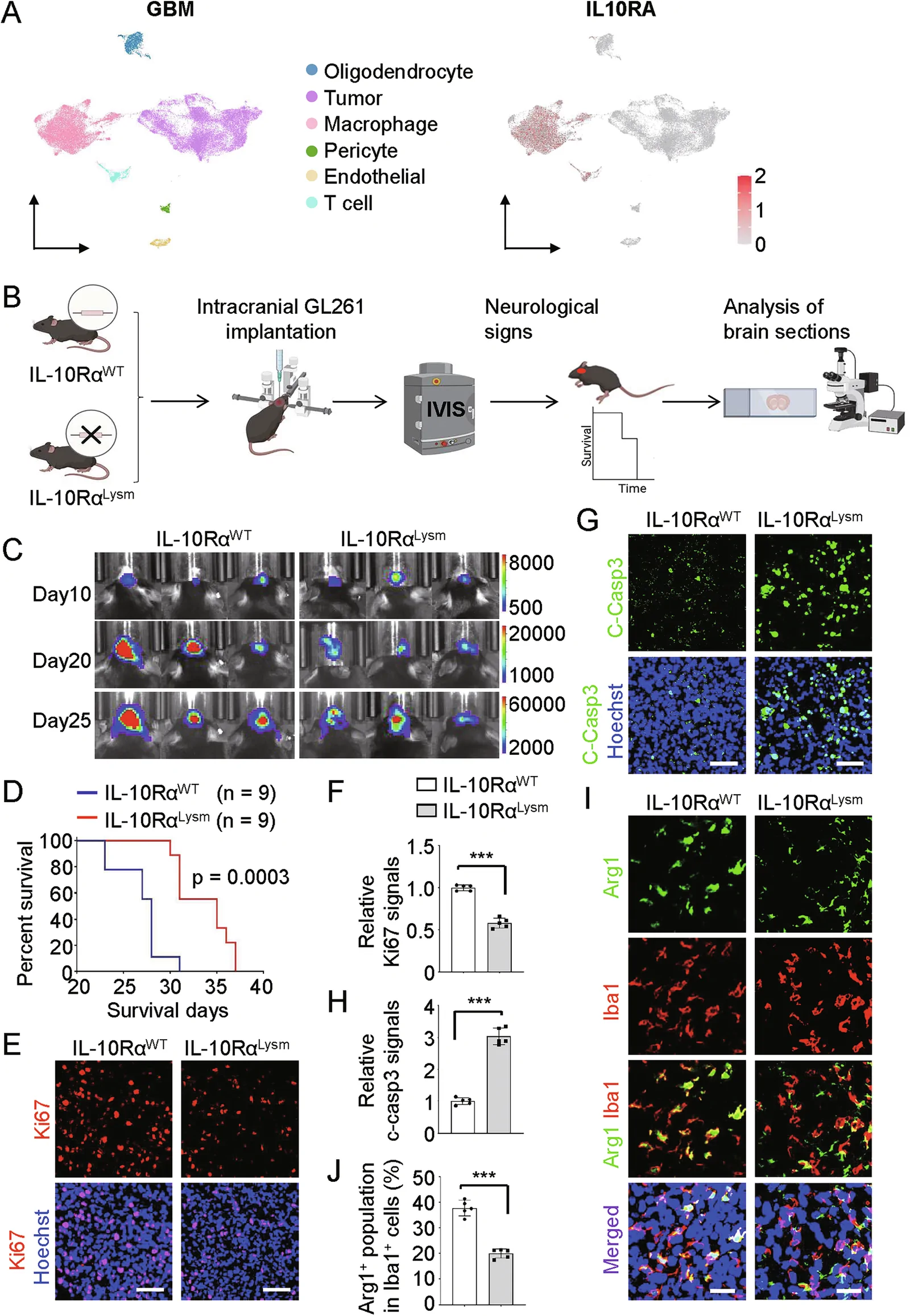
Myeloid knock-out of IL-10Rα inhibited GBM growth and tumor-supportive phenotypes of TAMs. **A** UMAP visualization of cell clusters in human GBM tissues (left) and UMAP plots showing the expression pattern of IL-10RA (right). Public 10× Genomics scRNA-Seq GEO datasets of 37742 cells from 13 cancer samples were preprocessed and integrated with R package Seurat using classical pipelines, followed by removal of batch effect with the Harmony algorithm. Unsupervised clustering was performed based on classical cell markers. Bar color represents gene expression level. **B** Workflow of construction and analysis of orthotopic GBMs in IL-10Rα^Lysm^ and wildtype mice. 5000 GL261 cells transduced with luciferase were implanted into the brains of IL-10Rα^Lysm^ and wildtype mice. Intracranial tumor growth was monitored through detection of bioluminescent signals with in vivo imaging system (IVIS). After appearance of neurological signs, tumor-bearing mice were sacrificed and tumor sections were subjected to afterward analysis. **C** In vivo bioluminescent images of intracranial tumor growth in IL-10Rα^Lysm^ and wildtype mice bearing GBM xenografts derived from GL261 cells. Five-thousand cells transduced with luciferase were implanted into mouse brains through intracranial injection. Representative bioluminescent images at the indicated days post-transplantation were shown. Tumor growth was attenuated in IL-10Rα^Lysm^ relative to wildtype mice. **D** Kaplan-Meier survival curves of tumor-bearing mice in (**C**). (*n* = 9 mice for each group; *P* = 0.0003; two-tailed log-rank test.). Representative images (**E**) and statistical quantification (**F**) of immunofluorescent analysis of Ki67 in intracranial GBMs in IL-10Rα^Lysm^ and wildtype mice. Frozen sections were immunostained with antibodies against Ki67, and counterstained with Hoechst to show nuclei. Tumors in IL-10Rα^Lysm^ mice had less Ki67 signals relative to those in wildtype mice. Scale bar, 80 μm. (*n* = 5 sections for each group; mean ± s.d.; *P* < 0.0001; ***P < 0.001; two-tailed unpaired student’s t-test). Representative images (**G**) and statistical quantification (**H**) of immunofluorescent analysis of cleaved caspase-3 in intracranial GBMs in IL-10Rα^Lysm^ and wildtype mice. Frozen sections were immunostained with antibodies against cleaved caspase-3, and counterstained with Hoechst to show nuclei. Tumors in IL-10Rα^Lysm^ mice had much more cleaved caspase-3 signals relative to those in wildtype mice. Scale bar, 80 μm. (*n* = 5 sections for each group; mean ± s.d.; *P* < 0.0001; ***P < 0.001; two-tailed unpaired student’s *t*-test). Representative images (**I**) and statistical quantification (**J**) of immunofluorescent analysis of the M2 macrophage marker Arg1 (green) and the pan-macrophage marker Iba1 (red) in intracranial GBMs in IL-10Rα^Lysm^ and wildtype mice. Frozen sections were immunostained with antibodies against Arg1 and Iba1, and counterstained with Hoechst to show nuclei. Tumors in IL-10Rα^Lysm^ mice had smaller proportions of Arg1^+^ macrophages relative to those in wildtype mice. Scale bar, 40 μm. (*n* = 5 sections for each group; mean ± s.d.; *P* < 0.0001 ***P < 0.001; two-tailed unpaired student’s *t*-test).

Macrophages in GBMs respond to several cytokines including IL-10, which functions by binding to the IL-10R complex comprised of the IL10-Rα and IL10-Rβ subunits [24]. Whereas IL10-Rα has a specific affinity to IL-10, IL10-Rβ is a component of multiple receptor complexes corresponding to IL-10 family members [25]. On the other hand, high expression of IL10-Rα has been observed in multiple types of cells in various tumors. We therefore utilized public single-cell RNA sequencing (scRNA-seq) data and evaluated IL10-Rα expression in different cell populations in GBMs [26, 27]. Based on classical cell markers, unsupervised clustering defined 37,742 cells including tumor cells (marked by EGFR, SOX2 and OLIG2), microglia/macrophages (marked by CD163, CD68 and LYZ), oligodendrocytes (marked by MAG, MBP and PLP1), endothelial cells (Marked by PECAM1, VWF and CLDN5), T cells (marked by CD3D, CD3E and CD8A), and pericytes (marked by PDGFR, RGS5 and GSPG4) (Fig. 1A, Fig. S1A). As expected, high expression of IL10-Rα was detected in macrophages (Fig. 1A). Thus, we chose IL10-Rα as the gene of interest to study IL-10 signaling in TAMs in GBMs.

To explore the impact of IL-10Rα-mediated signaling on macrophage differentiation and GBM development, we implanted GL261 cells and generated intracranial GBM in myeloid-specific IL-10Rα knockout (IL-10Rα^Lysm^) mice [17, 18] (Fig. 1B). TAMs in GBMs come from bone marrow-derived monocytes and brain-resident microglia that both belong to myeloid cells, and IL-10Rα was knocked out in both populations in the IL-10Rα^Lysm^ mice (Fig. S1B). GBM growth was much slower in IL-10Rα^Lysm^ relative to the wildtype mice as monitored by bioluminescent signals from tumor cells (Fig. 1C), resulting in a markedly extended survival of tumor-bearing mice (Fig. 1D). Immunofluorescent analysis of GBM sections showed that tumor cells had reduced proliferation as marked by Ki67 in the IL-10Rα^Lysm^ relative to the wildtype mice (Fig. 1E, F). Meanwhile, these tumors had elevated apoptosis as represented by the cleaved caspase-3 staining (Fig. 1G, H). These results demonstrated that disruption of IL-10Rα signaling in myeloid cells inhibited GBM growth. We then determined the effects of IL-10Rα knockout on TAMs. Immunofluorescent staining detected a remarkable reduction of the Arg1^+^ TAMs in GBMs in IL-10Rα^Lysm^ mice, whereas total macrophages marked by Iba1 had negligible changes (Fig. 1I, J). Meanwhile, there was an increase in the staining of the M1 macrophage marker MHCII (Fig. S1C–E). However, neither T cell infiltration nor vascularization in GBM tumors was not affected by IL-10Rα knockout in myeloid cells (Fig. S1F–I). Taken together, myeloid-specific knock-out of IL-10Rα inhibited GBM growth and affected TAM phenotypes.

### IL-10 production from TAMs was not affected by IL-10Rα deficiency

**Fig. 2:**
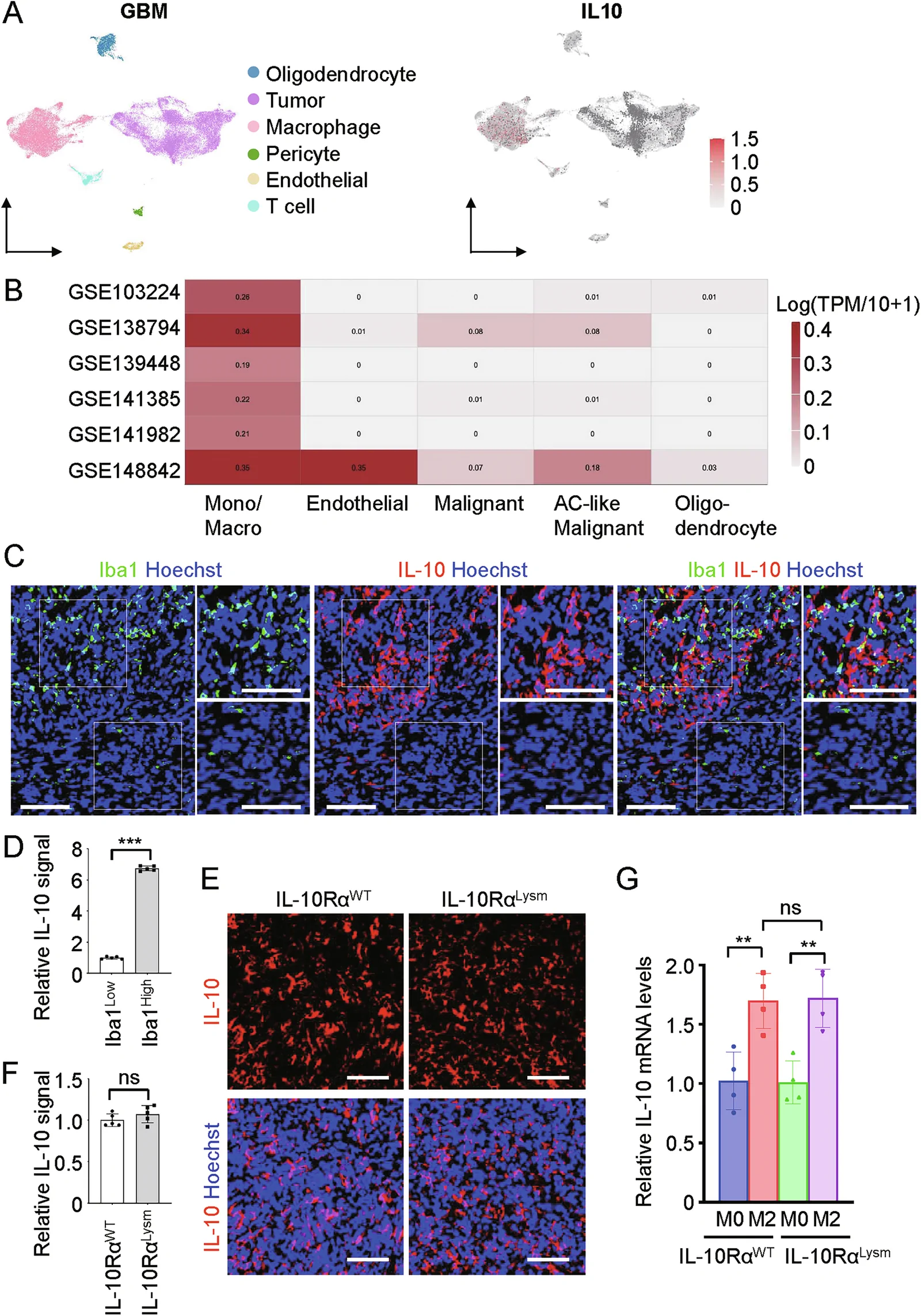
IL-10 production from TAMs was not affected by IL-10Rα deficiency. **A** UMAP visualization of cell clusters in human GBM tissues (left) and UMAP plots showing the expression pattern of IL10 (right). Bar color represents gene expression level. **B** Heatmap showing IL10RA expression in different cell types in GBMs. scRNA-seq datasets from GEO database (GSE103224, GSE138794, GSE139448, GSE141385, GSE141982, and GSE148842) was analyzed and visualized using the TISCH2 online module. The horizontal axis represents the cell types, and the vertical axis stands for the scRNA-seq datasets. Highest expression of IL10RA was observed in monocyte/macrophage populations. Color intensity represents average gene expression level calculated by log(TPM/10 + 1). Representative images (**C**) and statistical quantification (**D**) of immunofluorescent analysis of IL10 (red) and Iba1 (green) in intracranial mouse GBMs. Frozen sections were immunostained with antibodies against IL10 and Iba1, and counterstained with Hoechst to show nuclei. Much more IL10 signals were detected in tumor areas with high intensity of Iba1^+^ macrophages. Scale bar, 90 μm. (*n* = 5 sections for each group; mean ± s.d.; *P* < 0.0001; ***P < 0.001; two-tailed unpaired student’s *t*-test). Representative images (**E**) and statistical quantification (**F**) of immunofluorescent analysis of IL10 in intracranial GBMs in IL-10Rα^Lysm^ and wildtype mice. Frozen sections were immunostained with antibodies against IL10, and counterstained with Hoechst to show nuclei. Similar intensities of IL10 signals were observed in tumors in IL-10Rα^Lysm^ and wild-type mice. Scale bar, 40 μm. (*n* = 5 sections for each group; mean ± s.d.; *P* = 0.2496; ns, *P* > 0.05; two-tailed unpaired student’s *t*-test). **G** Quantitative PCR analysis of IL10 in bone marrow-derived macrophages from IL-10Rα^Lysm^ and wild-type mice upon cytokine induction. Bone marrow cells from mouse femurs were stimulated with 20 ng/ml M-CSF for 5 days for differentiation into the M0-like subtype. Alternatively, the M0-like macrophages were further stimulated with 20 ng/ml IL-4 and 20 ng/ml IL-10 for another 3 days to induce the M2-like subtype. IL10 expression was elevated in the M2- relative to M0-like subtype in both IL-10Rα^Lysm^ and wildtype macrophages. (*n* = 4 biological repeats; mean ± s.d.; *p* = 0.0071 for M2 vs. M0 in IL-10Rα^WT^; *p* = 0.0045 for M2 vs. M0 in IL-10Rα^Lysm^; *p* = 0.901 for IL-10Rα^WT^ vs. IL-10Rα^Lysm^ for M2 induction; **P < 0.01; ns, *P* > 0.05; two-tailed unpaired student’s t-test).

As the only known ligand for IL-10Rα, IL-10 has been detected in various cell types in GBMs [28, 29]. Interestingly, it is proposed that IL-10 stimulation may induce the M2 phenotype of TAMs that have elevated IL-10 production [29, 30]. We therefore investigated the potential impact of myeloid-specific IL-10Rα knock-out on IL-10 levels in GBM microenvironment. Analysis of the public scRNA-seq data showed that macrophages in GBM were the main source of IL-10 (Fig. 2A). Likewise, analysis with the Tumor Immune Single-cell Hub 2 (TISCH2), an online module that applied an independent and unified data processing of public scRNA-seq datasets, confirmed that macrophages were among the cell types with highest expression of IL-10 in glioma [31] (Fig. 2B). Furthermore, immunofluorescent staining of mouse GBM sections showed that IL-10 signals were co-enriched with TAMs marked by Iba1 (Fig. 2C, D). Whereas these data underscored the importance of TAMs in production of IL-10 in GBM microenvironment, surprisingly, total IL-10 levels in GBM tumors had negligible changes in IL-10Rα^Lysm^ relative to wildtype mice (Fig. 2E, F). In line with these observations, when bone-marrow cells were isolated from IL-10Rα^Lysm^ and wild-type mice and stimulated with IL-4 and IL-10 in vitro to induce the M2-like phenotype, the resultant M2-like macrophages had similar IL-10 levels with or without IL-10Rα disruption (Fig. 2G). These results suggest that production of IL-10 by TAMs in GBMs is not under the control of IL-10Rα signaling.

### RNA sequencing identified COX-1/2 as downstream targets of IL-10Rα in macrophages

**Fig. 3:**
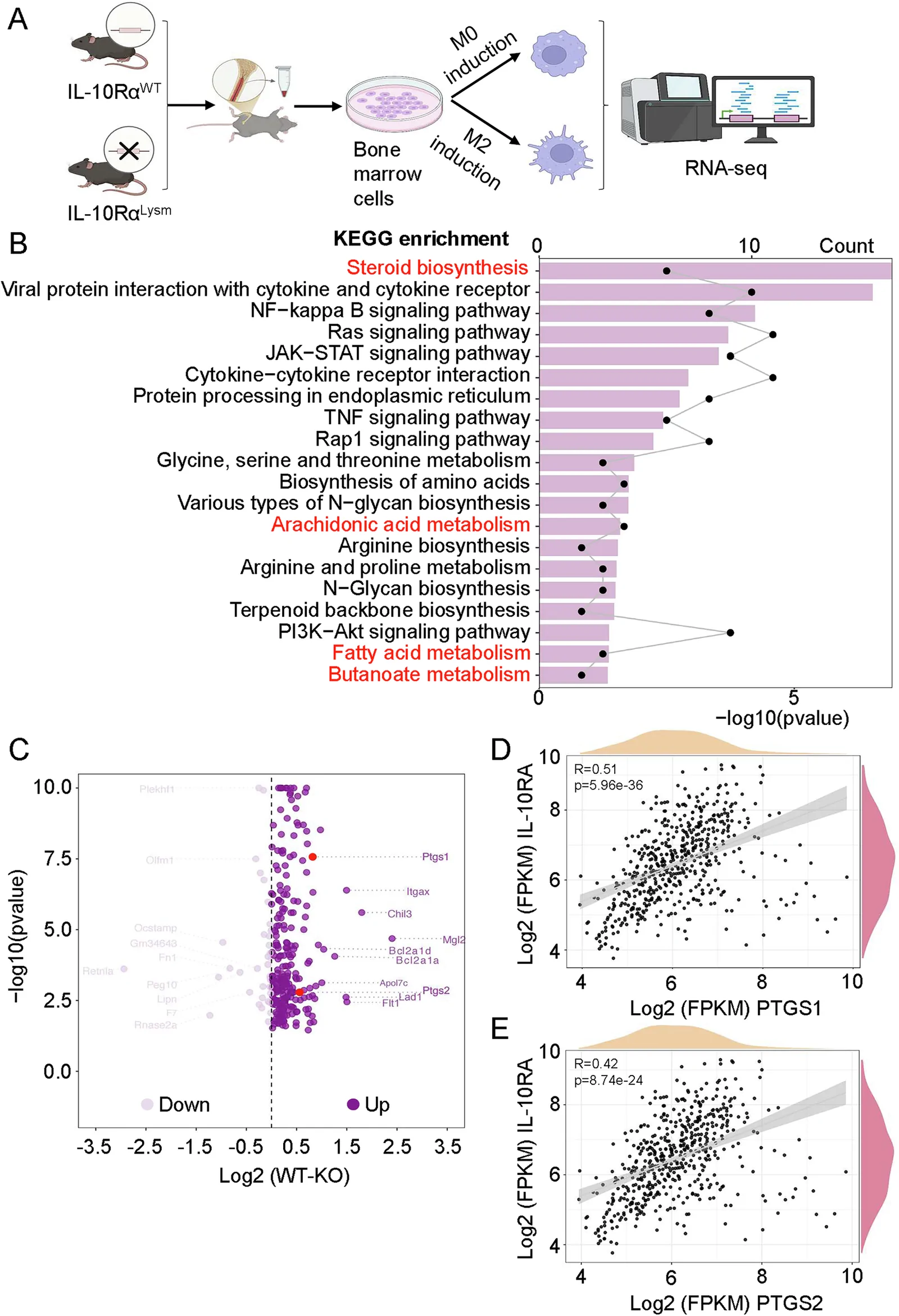
RNA sequencing identified COX-1/2 as downstream targets of IL-10Rα in macrophages. **A** Workflow of RNA-seq analysis of macrophages from IL-10Rα^Lysm^ and wildtype mice. Bone marrow cells from mouse femurs were stimulated with cytokines to induce M0- and M2-like subtype macrophages, followed by RNA sequencing. **B** KEGG pathway enrichment of genes differentially expressed in macrophages from IL-10Rα^Lysm^ and wildtype mice. Genes that showed log₂FC > 1 in M2-like compared to M0-like macrophages from wildtype mice and log₂FC > 0 in M2-like compared to M0-like macrophages from IL-10Rα^Lysm^ mice in the above RNA-seq were picked up. The genes with attenuated upregulation upon induction of M2-like macrophages from IL-10Rα^Lysm^ relative to wild-type mice were further subjected to KEGG analysis, which highlighted the enrichment of pathways relevant to lipid metabolism (in red). **C** Volcano plot of the genes with most remarkable differential expression in M2- relative to M0-like macrophages derived from IL-10Rα^Lysm^ mice. The DEGs with log₂FC > 1 in M2-like compared to M0-like macrophages from IL-10Rα^Lysm^ mice and log₂FC > 0 in M2-like compared to M0-like macrophages from wildtype mice were picked for analysis. Genes were ordered according to the extent of upregulated expression in M2- compared to M0-like subtypes in wildtype relative to IL-10Rα^Lysm^ macrophages. The Y axis represents *p* values of the upregulated gene expression in M2- compared to M0-like subtypes either in wild-type or IL-10Rα^Lysm^ macrophages. The X axis represents the extent of upregulated expression in M2- compared to M0-like subtypes in wildtype relative to IL-10Rα^Lysm^ macrophages. Pearson correlation analyses of IL10RA and PTGS1 (**D**) or PTGS2 (**E**) in GBM patients in TCGA database. Transcriptional data of GBM patients (*n* = 528) were analyzed with the *ggpubr* packages. IL10RA showed positive correlation with either PTGS1 and PTGS2.

Knowing that myeloid-specific knockout of IL-10Rα inhibited GBM growth and affected polarization of TAMs, we sought to find targetable downstream mediators of IL-10Rα signaling. To this end, bone-marrow cells were obtained from femurs of IL-10Rα^Lysm^ and wildtype mice, stimulated for M0- or M2-like differentiation, and subjected to RNA sequencing (RNA-seq) for analysis of the transcriptional changes during polarization of macrophages (Fig. 3A). Differentially expressed genes (DEGs) between M0- and M2-like macrophages were obtained, which revealed several genes either commonly upregulated or downregulated in cells derived from IL-10Rα^Lysm^ and wildtype mice (Fig. S2A). However, IL10-Rα deficiency did attenuate the upregulation of canonical M2 marker genes (Fig. S2B), indicating its important roles in the M2 polarization of macrophages. We focused on the genes that were markedly upregulated in the wildtype but not IL-10Rα^Lysm^ cells upon induction of M2-like phenotypes and performed gene enrichment analysis (Fig. S2C). As expected, KEGG and GO enrichment analyses discovered several established pathways in classical IL10-Rα signaling and macrophage M2-polarization (Fig. 3B, Fig. S2D). Because the JAK-STAT pathway, a well-known downstream mediator of IL-10Rα signaling, was revealed by the gene enrichment (Fig. 3B), STAT3 activation in TAMs was investigated in GBMs in IL-10Rα^Lysm^ and wild-type mice. Immunofluorescent staining detected much less pSTAT3 signals in TAMs in tumors from IL-10Rα^Lysm^ relative to wild-type mice (Fig. S3A and S3B), confirming the reliability of the RNA-seq analysis. Interestingly, the gene enrichment analyses underscored multiple pathways involved in lipid metabolism (Fig. 3B, Fig. S2D), suggesting the importance of metabolic regulation in macrophage differentiation. Meanwhile, when DEGs with remarkable increase in the wildtype but not IL-10Rα^Lysm^ cells upon M2 induction were ranked, *Ptgs1* and *Ptgs2* coding for the cyclooxygenases COX-1/2 with essential roles in lipid metabolism emerged among the top candidates (Fig. 3C). COX-1/2 are key enzymes that execute the conversion of arachidonic acid into the peroxide prostaglandin G2 and deeply participate in lipid metabolism [32]. Further analysis of the RNA-seq data demonstrated positive correlations between IL10RA and both PTGS1 and PTGS2 at the mRNA level (Fig. 3D, E). Moreover, scRNA-seq datasets from multiple human GBM samples indicated that high expression of both PTGS1 and PTGS2 were mainly observed in macrophages (Fig. S3C–E). Taken together, our RNA-seq analysis highlighted COX-1/2 as potential downstream mediators of IL-10Rα signaling and the consequent macrophage polarization.

### Disruption of IL-10Rα decreased COX-1/2 expression in TAMs

**Fig. 4:**
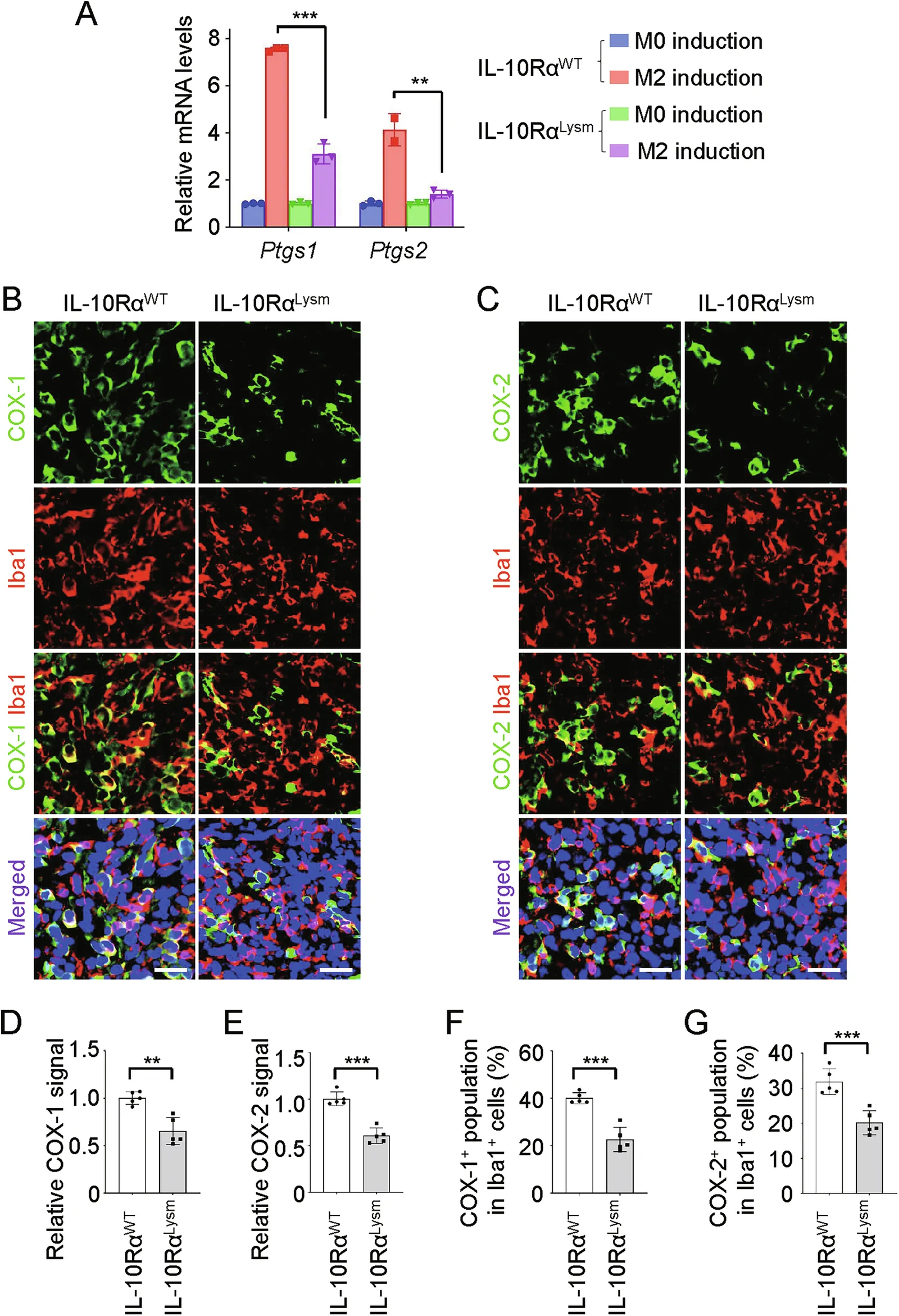
Disruption of IL-10Rα decreased COX-1/2 expression in TAMs. **A** Quantitative PCR analysis of *Ptgs1* (COX-1) and *Ptgs2* (COX-2) in bone marrow derived macrophages from IL-10Rα^Lysm^ and wild-type mice upon cytokine induction. Bone marrow cells from mouse femurs were stimulated into M0- and M2-like subtype macrophages. Both *Ptgs1* and *Ptgs2* expression was elevated in the M2- relative to M0-like subtype in both IL-10Rα^Lysm^ and wildtype macrophages. Yet the elevated expressions were much lower in IL-10Rα^Lysm^ relative to wild-type macrophages. (*n* = 3 biological repeats; mean ± s.d.; for M2 in IL-10Rα^Lysm^ vs. IL-10Rα^WT^ macrophages, *P* < 0.0001 for *Ptgs1*, *P* = 0.0056 for *Ptgs2*; ***P < 0.001; **P < 0.01; two-tailed unpaired student’s *t*-test). Representative images of immunofluorescent staining of COX-1 (**B**) and COX-2 (**C**) on TAMs in intracranial GBMs in IL-10Rα^Lysm^ and wild-type mice. Frozen sections were immunostained with antibodies against COX-1 or COX-2 along with Iba1, and counterstained with Hoechst to show nuclei. Scale bar, 40 μm. Statistical quantification of immunofluorescent analysis of COX-1 (**D**) and COX-2 (**E**) expression in intracranial GBMs in IL-10Rα^Lysm^ and wild-type mice. Remarkable reductions of COX-1 and COX-2 signals were detected in tumors in IL-10Rα^Lysm^ relative to wild-type mice. (*n* = 5 sections for each group; mean ± s.d.; *P* = 0.0011 for COX-1; *P* < 0.0001 for COX-2; **P < 0.01; ***P < 0.001; two-tailed unpaired student’s *t*-test). Statistical quantification of immunofluorescent analysis of COX-1 (**F**) and COX-2 (**G**) expression on TAMs in intracranial GBMs in IL-10Rα^Lysm^ and wildtype mice. Remarkable reductions in the COX-1^+^ and COX-2^+^ populations in Iba1^+^ TAMs were observed in tumors in IL-10Rα^Lysm^ relative to wild-type mice. (*n* = 5 sections for each group; mean ± s.d.; *P* = 0.0001 for COX-1; *P* < 0.0001 for COX-2; ***P < 0.001; two-tailed unpaired student’s *t*-test).

We then sought to validate if COX-1/2 were at the downstream of IL-10Rα signaling in TAMs. We initially checked the mRNA levels of COX-1/2 in macrophages derived from IL-10Rα^Lysm^ and wild-type mice upon cytokine stimulation. qPCR analysis showed that whereas IL-4/IL-10 induction dramatically elevated COX-1/2 expression in cells from wild-type mice, the elevation of COX-1/2 was markedly lower in cells from IL-10Rα^Lysm^ mice (Fig. 4A), suggesting that IL-10Rα signaling may activate COX-1/2 transcription. We further determined COX-1/2 expression in macrophages in GBM tissues from IL-10Rα^Lysm^ and wild-type mice. Immunofluorescent staining detected a remarkable reduction of COX-1/2 signals in GBMs in IL-10Rα^Lysm^ mice (Fig. 4B–E). Importantly, whereas a substantial proportion of tumor-infiltrating macrophages showed COX-1/2 expression in wildtype mice, the percentage of macrophages with COX-1/2 staining markedly decreased in GBMs in IL-10Rα^Lysm^ mice (Fig. 4F, G). Therefore, disruption of the IL-10α signaling did reduce COX-1/2 expression in macrophages in GBMs.

### Pharmacological inhibition of COX-1/2 suppressed pro-tumor phenotypes of macrophages

**Fig. 5:**
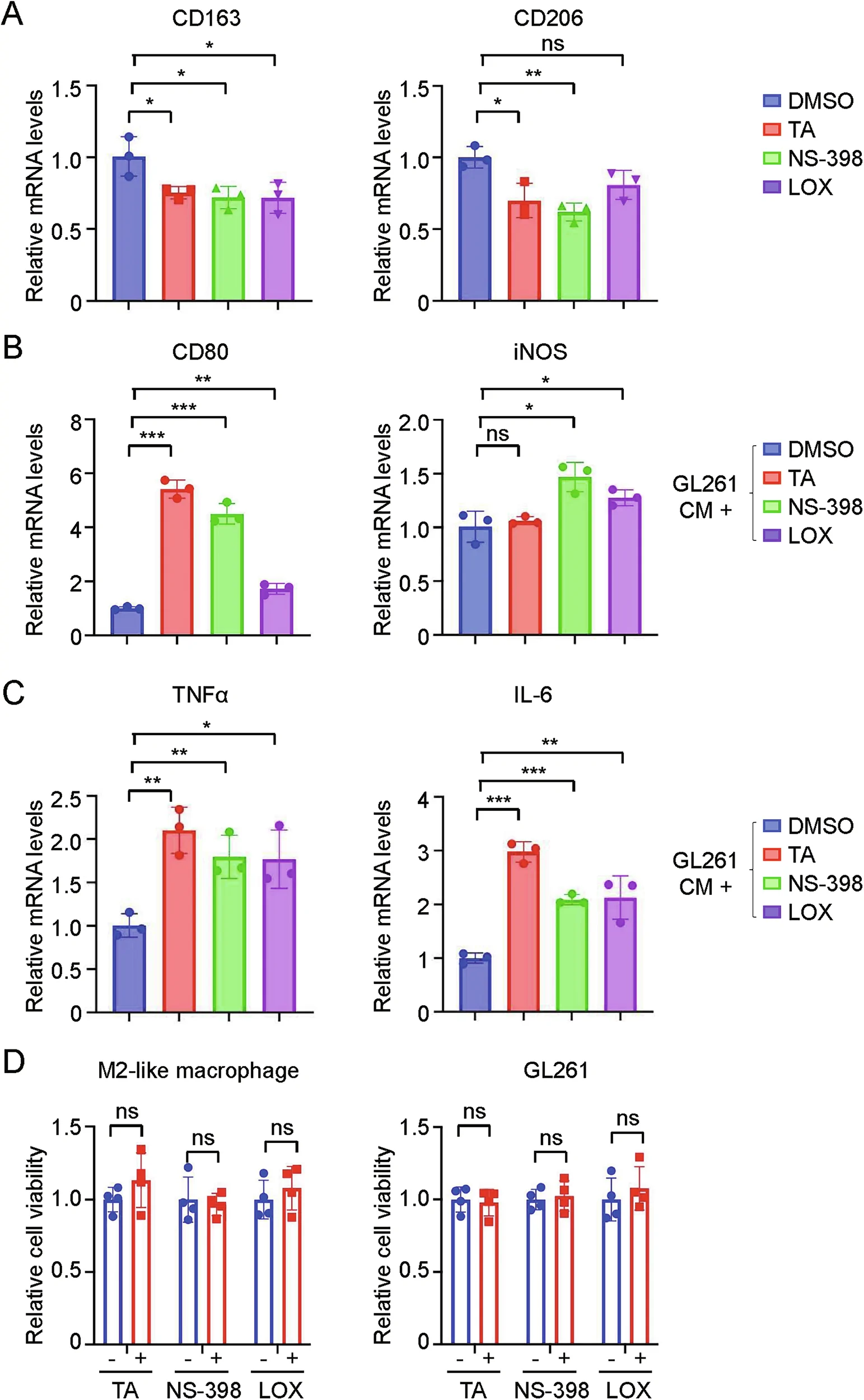
Pharmacological inhibition of COX-1/2 suppressed pro-tumor phenotypes of macrophages. **A** Quantitative PCR analysis of expressions of the M2-subtype macrophage markers CD163 and CD206 in mouse bone marrow derived macrophages in response to cyclooxygenase inhibitors. Bone marrow cells from mouse femurs were stimulated with M-CSF for 8 days for differentiation into M0-like macrophages, followed by treatment with Loxoprofen (5 μg/ml), NS-398 (5 μg/ml), or Tolfenamic Acid (10 μM) dissolved in DMSO for 24 h. Treatment with the cyclooxygenase inhibitors suppressed the expression of CD163 and CD206. (*n* = 3 biological repeats; mean ± s.d.; for CD163, *P* = 0.0391 for TA vs. DMSO, *P* = 0.0353 for NS-398 vs. DMSO, *P* = 0.0469 for LOX vs. DMSO；for CD206, *P* = 0.0208 for TA vs. DMSO, *P* = 0.0026 for NS-398 vs. DMSO, *P* = 0.0562 for LOX vs. DMSO; *P < 0.05; ns, *P* > 0.05; two-tailed unpaired student’s *t*-test). **B** Quantitative PCR analysis of expressions of the M1-subtype macrophage markers CD80 and iNOS in mouse bone marrow derived macrophages in response to cyclooxygenase inhibitors and conditioned medium from GL261. Bone marrow cells from mouse femurs were stimulated with M-CSF for 8 days for differentiation into M0-like macrophages, followed by treatment with the COX-1/2 inhibitors for 24 h. The cells were then cultured in GL261 conditioned medium along with the COX-1/2 inhibitors for another 24 h. Treatment with the cyclooxygenase inhibitors elevated the expression of CD80 and iNOS. (*n* = 3 biological repeats; mean ± s.d.; for CD80, *P* < 0.0001 for TA vs. DMSO, *P* < 0.0001 for NS-398 vs. DMSO, *P* = 0.0039 for LOX vs. DMSO; for iNOS, *P* = 0.5564 for TA vs. DMSO, *P* = 0.0156 for NS-398 vs. DMSO, *P* = 0.0454 for LOX vs. DMSO; ***P < 0.001; **P < 0.01; *P < 0.05; ns, *P* > 0.05; two-tailed unpaired student’s *t*-test). **C** Quantitative PCR analysis of expressions of the inflammatory cytokines TNFα and IL-6 in mouse bone marrow derived macrophages in response to cyclooxygenase inhibitors and conditioned medium from GL261. Treatment with the cyclooxygenase inhibitors elevated the expression of TNFα and IL-6. (*n* = 3 biological repeats; mean ± s.d.; for TNFα, *P* = 0.0032 for TA vs. DMSO, *P* = 0.0085 for NS-398 vs. DMSO, *P* = 0.0221 for LOX vs. DMSO; for IL-6, *P* < 0.0001 for TA vs. DMSO, *P* = 0.0001 for NS-398 vs. DMSO, *P* = 0.0092 for LOX vs. DMSO; ***P < 0.001; **P < 0.01; *P < 0.05; two-tailed unpaired student’s *t*-test). **D** Cell viability of M2-like macrophages (left) and GL261 cells (right) upon treatment with cyclooxygenase inhibitors. Bone marrow cells from mouse femurs were induced with cytokines to differentiated into the M2-like macrophages. 2000 ma, and cell viability was determined by CCK8 assays. Treatment with cyclooxygenase inhibitors had negligible effects on cell viability (*n* = 4 biological repeats; mean ± s.d.; ns, *P* > 0.05; two-tailed unpaired student’s t-test).

As pivotal metabolic enzymes, COX-1/2 have several small molecular inhibitors. We tested the potential impacts of COX-1/2 inhibitors on macrophage phenotypes. Mouse bone-marrow cells were initially primed with M-CSF and then treated with the COX-1/2 inhibitors Loxoprofen (LOX), NS-398, or Tolfenamic acid (TA). qPCR analysis showed that all the three COX-1/2 inhibitors suppressed the expression of the M2-subtype marker CD163, whereas TA and NS-398 inhibited another M2 marker CD206 (Fig. 5A). However, treatment with the COX-1/2 inhibitors with such settings hardly affected expression of TNFα and CD80, two genes preferentially expressed in M1-subtype macrophages (Fig. S4A). Alternatively, the primed bone-marrow cells were exposed to the conditioned medium from GL261 cells at the presence of the COX-1/2 inhibitors. qPCR analysis of the treated cells showed that all the COX-1/2 inhibitors elevated the expression of the M1-subtype marker CD80, whereas NS-398 and LOX increased another M1 marker iNOS (Fig. 5B). Interestingly, while macrophages may produce several kinds of cytokines, treatment with the COX-1/2 inhibitors upregulated the levels of TNFα and IL-6, two well-established inflammatory factors (Fig. 5C). Of note, under such conditions, the COX-1/2 inhibitors did not universally suppress expression of the M2 markers CD163 and CD206 in macrophages, although NS-398 and LOX treatments caused decreases in CD206 and CD163, respectively (Fig. S4B). Meanwhile, treatment with the above COX-1/2 inhibitors showed negligible impact on the viability of either M2-like macrophages or GL261 cells in vitro (Fig. 5D). Taken together, pharmacological inhibition of COX-1/2 may induce the switch of macrophages from pro-tumor to anti-tumor phenotypes without severe impact on cell viability.

### Treatment with the COX-1/2 inhibitor NS-398 mitigated orthotopic GBM growth

**Fig. 6:**
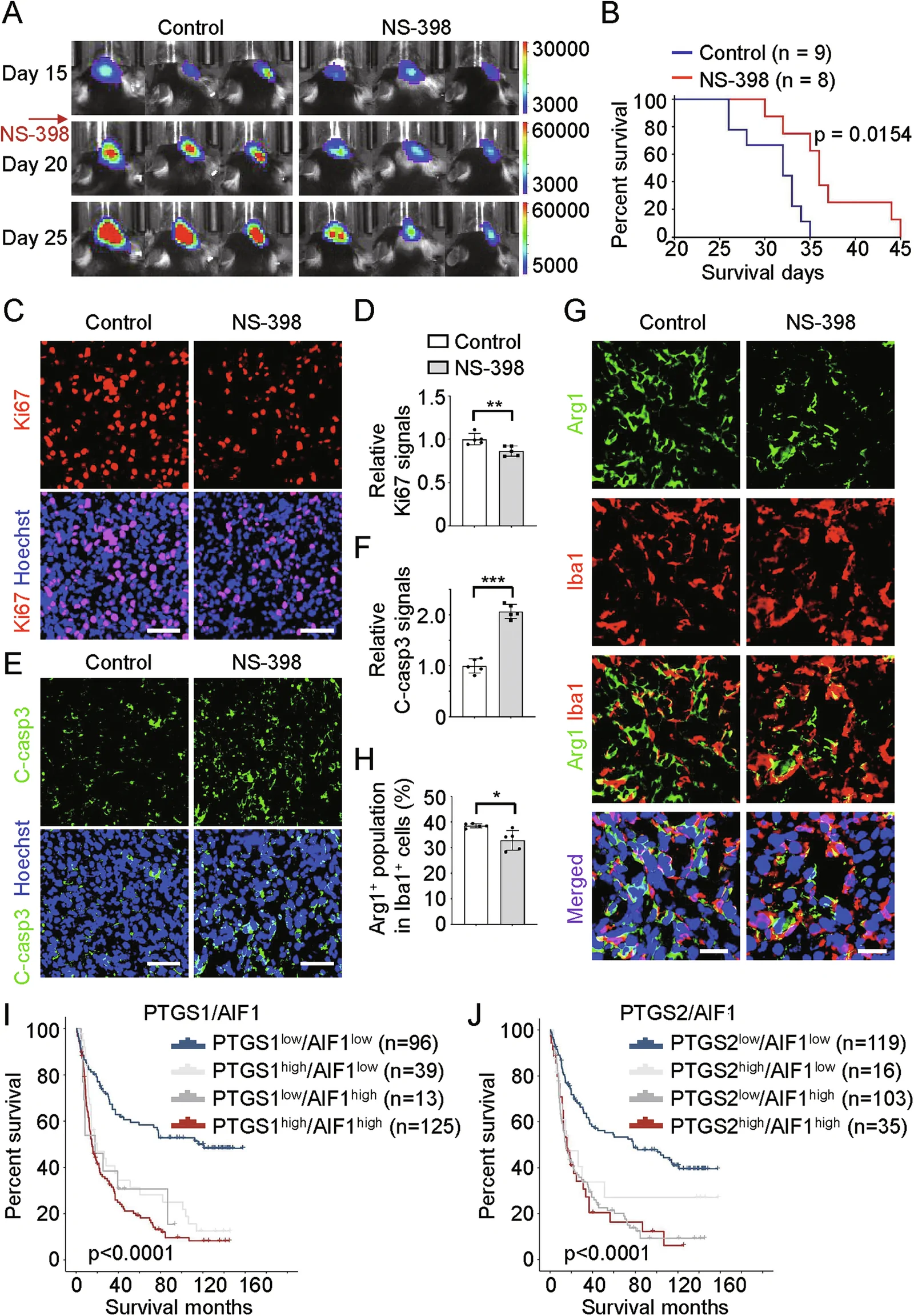
Treatment with the COX-1/2 inhibitor NS-398 mitigated orthotopic GBM growth. **A** In vivo bioluminescent images of intracranial tumor growth in mice bearing GL261-derived GBM xenografts upon NS-398 treatment. Five-thousand cells transduced with luciferase were implanted into mouse brains through intracranial injection. Fifteen days post tumor implantation, tumor-bearing mice were treated with NS-398 (10 mg/kg, 100 μl in volume) through intraperitoneal injection every day until the demonstration of neurological signs. Representative bioluminescent images at the indicated days post-transplantation were shown. Tumor growth was mitigated after NS-398 treatment. **B** Kaplan-Meier survival curves of tumor-bearing mice in (**A**). (*n* = 9 mice for the control group; *n* = 8 mice for the NS-398 treatment group; *P* = 0.0154; two-tailed log-rank test.). Representative images (**C**) and statistical quantification (**D**) of immunofluorescent analysis of Ki67 in intracranial GBMs treated with control or NS-398. Frozen sections were immunostained with antibodies against Ki67, and counterstained with Hoechst to show nuclei. NS-398 relative to the control treatment reduced Ki67 signals in GBMs. Scale bar, 80 μm. (*n* = 5 sections for each group; mean ± s.d.; *P* = 0.0078; **P < 0.01; two-tailed unpaired student’s *t*-test). Representative images (**E**) and statistical quantification (**F**) of immunofluorescent analysis of cleaved caspase-3 in intracranial GBMs treated with control or NS-398. Frozen sections were immunostained with antibodies against cleaved caspase-3, and counterstained with Hoechst to show nuclei. NS-398 relative to the control treatment increased cleaved caspase-3 signals in GBMs. Scale bar, 80 μm. (*n* = 5 sections for each group; mean ± s.d.; *P* < 0.0001; ***P < 0.001; two-tailed unpaired student’s *t*-test). Representative images (**G**) and statistical quantification (**H**) of immunofluorescent analysis of Arg1 (green) and Iba1 (red) in intracranial GBMs treated with control or NS-398. Frozen sections were immunostained with antibodies against Arg1 and Iba1, and counterstained with Hoechst to show nuclei. NS-398 relative to the control treatment reduced the proportions of Arg1^+^ macrophages in GBMs. Scale bar, 40 μm. (*n* = 5 sections for each group; mean ± s.d.; *P* = 0.0124; *P < 0.05; two-tailed unpaired student’s *t*-test). Combined prognostic impact of the macrophage marker gene AIF1 and PTGS1 (**I**) or PTGS2 (**J**) and on survival of glioma patients in the CGGA database. Patients were divided into four groups according to the expression levels of PTGS1/2 and AIF1, while the optimal cutoff point was automatically determined by *surv_cutpoint*. Kaplan–Meier survival analysis showed that patients with simultaneous high expression of PTGS1/2 and AIF1 had the worst survival, whereas those with simultaneous low expression showed the best survival. (*P* < 0.0001 for PTGS1/2^High^AIF1^High^ vs. PTGS1/2^Low^AIF1^Low^; two-tailed log-rank test.).

Whereas treatments with all the three tested COX-1/2 inhibitors reduced the levels of M2 markers in macrophages upon IL-4/IL-10 stimulation in vitro, NS-398 was able to penetrate the blood-brain barrier (BBB) [33], and thus we tested the capacity of NS-398 in treating intracranial GBMs in mice. Bioluminescent imaging demonstrated that NS-398 treatment delayed growth of orthotopic GBMs (Fig. 6A, Fig. S5A), resulting in a notably extended survival of tumor-bearing mice (Fig. 6B). Immunofluorescent analysis of the GBM sections showed that NS-398 treatment suppressed cell proliferation marked by Ki67, but increased cell apoptosis according to cleaved caspase-3 signals (Fig. 6C–F). Moreover, immunofluorescent staining detected reduced proportions of the Arg1^+^ TAMs in GBMs upon NS-398 relative to control treatment (Fig. 6G, H), whereas total macrophages marked by Iba1 had negligible changes (Fig. S5B). Meanwhile, there was an increase in the staining of the M1 marker MHCII on macrophages (Fig. S5C, D). These data strongly suggested that NS-398 treatment suppressed pro-tumor phenotypes of macrophages and mitigated GBM growth. Last but not least, we tried to determine the clinical implications of COX-1/2 expression on macrophages in prognosis of glioma patients. We took advantage of the Chinese Glioma Genome Atlas (CGGA) database and categorized patients into groups according to the expression levels of COX-1/2 and AIF1 (Iba1). Not surprisingly, the patients with high expression of both COX-1/2 and AIF1 had a worse survival, whereas those with COX-1/2 and AIF low expressions had a better survival (Fig. 6I, J). In summary, these results indicated the clinical potential of inhibiting COX-1/2 in macrophages for GBM treatment.

## Discussion

This study utilized orthotopic xenografts in mice with myeloid-specific knockout of *Il10ra* and showed that the IL-10Rα-COX-1/2 axis promoted the tumor-supportive roles of TAMs in GBMs. The anti-tumor effects of either IL-10Rα knockout or COX-1/2 inhibition were credited to the phenotype changes of macrophages, which was based on the observation of the reduced expression of M2 markers along the elevated levels of M1 markers. However, these phenomena did not necessarily stand for a total switch of TAMs from M2 to M1 subtype. Whereas IL-10 was widely reported as a cytokine produced by the M2 TAMs [29, 34], knock-out of IL-10Rα showed negligible influence on IL-10 expression in macrophages in GBMs (Fig. 2). In fact, although TAMs are often roughly categorized into the tumor-supportive M2 and the tumor-suppressive M1 subtypes, they may be composed of a spectrum of intermediate differentiation status [35]. On the other hand, inhibition of the IL-10Rα-COX-1/2 axis elevated expression of multiple effectors that may involve in the anti-tumor activities of macrophages, including CD80 and MHCII associated with antigen presentation (Fig. 5B, Fig. S5C, D), the pro-inflammatory cytokines TNFα and IL-6 (Fig. 5C), and iNOS with metabolic regulatory functions in cytotoxicity against tumor cells (Fig. 5B). Therefore, while our discoveries unraveled the critical roles of IL-10Rα signaling in endowing macrophages with tumor-supportive activities, the fate of macrophages after disruption of IL-10Rα signaling is worth further investigations.

The widely utilized LysM-Cre and the IL‑10Rα^flox/flox^ mouse strains were applied for myeloid-specific targeting of IL‑10Rα in this study. Although the strategy has been proven by several studies [17, 18], the LysM-Cre system may target several types of cells, including monocyte/macrophage, microglia, neutrophil, and even neuron [36]. Given that the intensities of neutrophils and neurons are quite low in glioma tissues, and that there are quite few literatures concerning IL-10 signaling in these cells, it is reasonable to use the LysM-Cre and the IL‑10Rα^flox/flox^ system in studying IL-10/IL-10Rα signaling in TAMs at the current stage. However, TAMs are composed of macrophages and microglia, and IL-10Rα has been reported to curb microglial responses and TNFα overproduction [37]. Interestingly, when TAMs were further stratified into monocyte-derived macrophages and microglia, bioinformatic analysis showed that both populations contained a substantial proportion of IL-10Rα^+^ cells along with a small part of IL-10^+^ cells (Fig. S6A–E). In contrast, analysis of a public scRNA-seq dataset suggested that microglia in tumor-free human brains almost had no IL-10 or IL-10Rα expression (Fig. S6F). These data indicated that IL-10/IL-10Rα signaling in microglia may be quite different with or without the presence of tumors. To distinguish macrophages and microglia in terms of IL-10/IL-10Rα signaling, it would be better to isolate microglia from glioma-bearing animals for mechanistic studies. However, isolation and differentiation of bone-barrow cells into different macrophage states have well-established protocols, whereas selective purification of microglia from tumor tissues and further induction of polarization are difficult at present. Moreover, immunostaining of intracranial GBMs showed that microglia were constrained in the peri-tumoral benign regions (Fig. S6G, H). Therefore, this study mainly used BMDM for mechanistic and phenotypic studies for TAMs, which may be verified in the future in microglia isolated from tumors.

RNA-seq of macrophages with or without *Il10ra* knockout, along with bioinformatic analysis of public transcriptomic datasets from human GBMs, indicated cyclooxygenases COX-1/2 as downstream mediators of IL-10Rα signaling that promoted macrophage differentiation into tumor-supportive phenotypes. Both COX-1 and COX-2 produce prostanoids that are active lipid mediators of inflammatory responses, yet COX-1 is widely present in most tissues, while COX-2 is primarily found at sites of inflammation under physiological conditions [38]. Previous reports showed that COX-2 was highly expressed in GBM relative to normal brain tissues [39]. Whereas some investigation suggested that COX-2 may promote tumor cell proliferation and resistance to irradiation, other studies indicated that COX-2 expression in tumor cells may function by regulating components in the tumor microenvironment such as microglia through the downstream product prostaglandin E2 (PGE2) [40, 41]. Interestingly, although PGE2 is primarily a pro-inflammatory factor, higher expression of COX-2 in GBMs is correlated with poorer survival, highlighting the complex roles of COX-2 in tumors [40]. Our bioinformatic data showed that monocytes/macrophages rather than tumor cells were the cell populations with the highest expressions of COX-1/2 (Fig. S3C–E). Hence, therapeutic benefits of targeting COX-1/2 in GBMs are likely accredited to the change of the tumor immune microenvironment. The relationship between COX-2 and IL-10 signaling is also complicated. Simultaneous upregulations of COX-2 and IL-10 were observed under various circumstances [42], indicating that they may have synergistic functions. In fact, COX-2 was reported to induce IL-10 expression via PGE2 [43]. On the other hand, IL-10 inhibited COX-2 induction upon LPS stimulation [44]. Anyway, our data showed that transcription of COX-1/2 was under the control of IL-10Rα signaling during macrophage differentiation into the tumor-supportive phenotype (Fig. 4A). Nevertheless, the signal transducers linking IL-10Rα and COX-1/2 transcription and the downstream effectors of COX-1/2 require further investigations.

GBM is featured by its immune suppressive microenvironment dominated by TAMs [6]. Targeting signaling pathways underlying TAM differentiation into tumor-supportive phenotypes such as CSF-1R has emerged as an effective strategy that inhibits GBM growth and promotes anti-tumor immune response [8, 45]. Our study showed that IL-10Rα signaling in macrophages was an effective target in GBMs, which may be combined with immunotherapies to achieve maximum benefits. To our knowledge, there are no commercially available small molecular inhibitors that directly block IL-10Rα signaling. To make thing worse, antibody drugs may be hindered by the BBB for GBM treatment. Fortunately, we found that COX-1/2 were downstream mediators of the IL-10Rα signaling in macrophage differentiation, indicating that disruption of COX-1/2 may interfere IL-10Rα signaling in TAMs. Moreover, inhibitors for COX-1/2 effectively changed the phenotypes of macrophages in vitro (Fig. 5). Because COX-2 had been regarded as a key player either in tumors or in inflammation, most inhibitors were developed against COX-2 rather than COX-1. NS-398 is a well-established inhibitor for COX-2, although it may inhibit COX-1 at higher concentrations [46]. Since NS-398 has been reported to penetrate the BBB [33], we applied NS-398 for treatment of intracranial GBMs in mice and observed remarkable changes in TAMs in vivo (Fig. 6). Of note, non-steroidal anti-inflammatory drugs (NSAID) may act on both COXs and have the potential to pass the BBB [47]. Given that the two cyclooxygenases both had high expression in TAMs, simultaneous inhibition of COX-1/2 may have an even stronger impact on TAMs in GBMs, which warrants future trials.

## Supplementary information

Final version of supplementary information

Supplementary Figure S1

Supplementary Figure S2

Supplementary Figure S3

Supplementary Figure S4

Supplementary Figure S5

Supplementary Figure S6

Original data for qPCR analysis

## Data Availability

RNA-seq datasets have been deposited at the Genome Sequence Archive at the National Genomics Data Center (Beijing, China) that are accessible under BioProject CRA033338. This paper does not report original code.
