# Supplementary material for: Cyclooxygenase-mediated IL-10Rα signaling underlies pro-tumor phenotypes of macrophages in glioma
Source: Cell Death Dis. 2026 Jul 27;17(1):898. doi: 10.1038/s41419-026-09072-7 (PMC13686974; doi:10.1038/s41419-026-09072-7)
Supplement: Supplementary file 1 [file 41419_2026_9072_MOESM8_ESM.pdf]

Fig 2G

IL10	Foldchange
WT	1.119224928
WT	1.312666111
WT	0.902813565
WT	0.753928701
WT-M2	1.818186259
WT-M2	1.93522324
WT-M2	1.406880703
WT-M2	1.627322285
IL-10RA	0.885767519
IL-10RA	1.261377409
IL-10RA	0.867538687
IL-10RA	1.031683179
IL-10RA-M	1.938579634
IL-10RA-M	1.585568273
IL-10RA-M	1.911890635
IL-10RA-M	1.43893358

Fig 4A

Ptgs1	Foldchange	Ptgs2	Foldchange
WT-M0	1.01865581	WT-M0	1.107009
WT-M0	1.01161944	WT-M0	0.997692
WT-M0	0.970410231	WT-M0	0.905425
WT-M2	7.603515822	WT-M2	4.616075
WT-M2	7.447037729	WT-M2	3.344129
WT-M2	7.603515822	WT-M2	3.64689
IL10RA-M0	1.060880943	IL10RA-M0	1.029572
IL10RA-M0	0.982999617	IL10RA-M0	0.940856
IL10RA-M0	0.95611944	IL10RA-M0	1.029572
IL10RA-M2	2.966159324	IL10RA-M2	1.227207
IL10RA-M2	2.767524507	IL10RA-M2	1.390283
IL10RA-M2	3.576618131	IL10RA-M2	1.564149

Fig 5A

CD163	Foldchange	CD206	Foldchange
control	0.866537046	control	0.937354
control	1.143402487	control	1.084227
control	1.009284801	control	0.983957
TA	0.775572381	TA	0.601513
TA	0.703846792	TA	0.833161
TA	0.780966913	TA	0.66281
NS398	0.73883972	NS398	0.545884
NS398	0.634342247	NS398	0.66281
NS398	0.786398968	NS398	0.649169
LOX	0.854607174	LOX	0.886791
LOX	0.600124333	LOX	0.695762
LOX	0.73883972	LOX	0.844791

Fig 5B

iNOS	Foldchange	CD80	Foldchange
GL216	0.842841545	GL216	0.954842
GL216	1.112136086	GL216	0.981686
GL216	1.066832243	GL216	1.066832

TA+261	1.037659659	TA+261	5.749089
TA+261	1.044877153	TA+261	5.438969
TA+261	1.104454001	TA+261	5.074738
ns-398+26	1.313424556	ns-398+26	4.326899
ns-398+26	1.529789694	ns-398+26	4.935969
ns-398+26	1.561933827	ns-398+26	4.237852
LOX+261	1.175547906	LOX+261	1.883392
LOX+261	1.350349946	LOX+261	1.498307
LOX+261	1.30435207	LOX+261	1.781797

Fig 5C

tnf	Foldchange	IL-6	Foldchange
GL216	0.895025071	GL216	1.084227
GL216	0.965936329	GL216	0.899171
GL216	1.156688184	GL216	1.025741
TA+261	2.345669898	TA+261	3.131094
TA+261	2.143546925	TA+261	3.045474
TA+261	1.815038311	TA+261	2.763826
ns-398+26	1.635804117	ns-398+26	2.198724
ns-398+26	1.670175839	ns-398+26	2.037312
ns-398+26	2.084931522	ns-398+26	2.037312
LOX+261	2.158456473	LOX+261	2.356534
LOX+261	1.602139755	LOX+261	2.372925
LOX+261	1.547564994	LOX+261	1.666321

Fig S4A

TNFa	Foldchange	CD80	Foldchange
control	1.218083046	control	0.918069
control	0.826228366	control	1.084233
control	0.955688588	control	0.997698
TA	0.699604996	TA	0.730358
TA	0.897891169	TA	0.899176
TA	0.68047426	TA	0.821695
NS398	0.699604996	NS398	0.838961
NS398	0.685207333	NS398	0.766668
NS398	0.982556651	NS398	0.997698
LOX	0.8673057	LOX	0.930885
LOX	0.891688982	LOX	0.868546
LOX	0.910425263	LOX	0.816019

Fig S4B

CD206	Foldchange	CD80	Foldchange
GL216	0.849362567	GL216	0.876545
GL216	1.097676118	GL216	1.148618
GL216	1.052961314	GL216	0.974837
TA+261	1.269668995	TA+261	1.189124
TA+261	1.226419409	TA+261	1.156608
TA+261	1.192882906	TA+261	1.12498
ns-398+26	0.024424155	ns-398+26	0.784529
ns-398+26	0.032905041	ns-398+26	0.77911
ns-398+26	0.056895267	ns-398+26	0.757806
LOX+261	1.010068008	LOX+261	0.659708
LOX+261	0.923031913	LOX+261	0.365996
LOX+261	0.873241399	LOX+261	0.586377
