# Supplementary material for: Cyclooxygenase-mediated IL-10Rα signaling underlies pro-tumor phenotypes of macrophages in glioma
Source: Cell Death Dis. 2026 Jul 27;17(1):898. doi: 10.1038/s41419-026-09072-7 (PMC13686974; doi:10.1038/s41419-026-09072-7)
Supplement: Supplementary file 2 [file 41419_2026_9072_MOESM1_ESM.docx]

**Supplementary Figure Legends**

**Figure S1. Myeloid knock-out of IL-10Rα inhibited GBM growth and affected phenotypes of TAMs**

A. Dot plots showing the expression of the classical markers for each cell cluster identified in the scRNA-seq data of GBM tissues. Dot sizes represent the percentage of cells in the cluster expressing the indicated genes. Dot colors represent the normalized gene expression.
B. Analysis of the *Il10ra* knock-out status in myeloid and non-myeloid cells from the IL-10Rα^Lysm^ and wildtype mice. Monocyte-derived macrophages were obtained by M-CSF-induced differentiation of bone marrow cells from mouse femurs. Microglia were isolated from mouse brains through MACS sorting with anti-CD11b beads. Mouse tail tissues were used as non-myeloid cells. Genomic DNAs were extracted and subjected to PCR analysis. Deletion of the exon3 of the *Il10ra* gene resulted in a shift-down of PCR products, which was observed in both monocyte-derived macrophages and microglia but not in non-myeloid cells.

**C**-**E**. Representative images (**C**) and statistical quantifications (**D** and **E**) of immunofluorescent analysis of the M1 macrophage marker MHCII (green) and the pan-macrophage marker Iba1 (red) in intracranial GBMs in IL-10Rα^Lysm^ and wildtype mice. Frozen sections were immunostained with antibodies against MHCII and Iba1, and counterstained with Hoechst to show nuclei. Tumors in IL-10Rα^Lysm^ mice had larger proportions of MHCII^+^ macrophages relative to those in wildtype mice (**D**), while intensity of tumor-infiltrating Iba^+^ macrophages was reduced in IL-10Rα^Lysm^ relative to wildtype mice (**E**). Scale bar, 40 μm. (n = 5 sections for each group; mean ± s.d.; P = 0.0082; **, P < 0.01; two-tailed unpaired student’s t-test)

**F** and **G**. Representative images (**F**) and statistical quantification (**G**) of immunofluorescent analysis of the T cell marker CD3 in intracranial GBMs in IL-10Rα^Lysm^ and wildtype mice. Frozen sections were immunostained with antibodies against CD3, and counterstained with Hoechst to show nuclei. Similar intensities of CD3^+^ T cells were observed in tumors in IL-10Rα^Lysm^ and wildtype mice. Scale bar, 40 μm. (n = 5 sections for each group; mean ± s.d.; P = 0.4466; ns, P > 0.05; two-tailed unpaired student’s t-test)

**H** and **I**. Representative images (**H**) and statistical quantification (**I**) of immunofluorescent analysis of the endothelial cell marker CD31 in intracranial GBMs in IL-10Rα^Lysm^ and wildtype mice. Frozen sections were immunostained with antibodies against CD31, and counterstained with Hoechst to show nuclei. Similar intensities of CD31^+^ vessels were observed in tumors in IL-10Rα^Lysm^ and wildtype mice. Scale bar, 80 μm. (n = 5 sections for each group; mean ± s.d.; P = 0.862; ns, P > 0.05; two-tailed unpaired student’s t-test)

**Figure S2. RNA sequencing identified COX-1/2 as downstream targets of IL-10Rα in macrophages**

**A.** Venn diagram showing genes commonly upregulated or downregulated in M2- relative to M0-like macrophages derived from IL-10Rα^Lysm^ and wildtype mice.

**B.** Heatmap showing expression of multiple representative genes in bone marrow cells derived from IL-10Rα^Lysm^ and wildtype mice and induced for M0- and M2-like macrophage differentiation. Classical macrophage markers were labeled in red. Bar colors represent expression levels.

**C.** Workflow of the analysis of potential mediators at the downstream of IL10-Rα that regulate differentiation of macrophages. Genes that showed log₂FC > 1 in M2-like compared to M0-like macrophages from wildtype mice and log₂FC > 0 in M2-like compared to M0-like macrophages from IL-10Rα^Lysm^ mice in the RNA-seq were picked up. The genes with attenuated upregulation upon induction of M2-like macrophages from IL-10Rα^Lysm^ relative to wildtype mice were further subjected to were further subjected to functional enrichment analysis with KEGG and GO. The genes of interest were the ones that belonged to the enriched pathways and had marked changes in expression in M2-like macrophages derived from IL-10Rα^Lysm^ relative to these from wildtype mice.

**D**. GO pathway enrichment of genes differentially expressed in macrophages from IL-10Rα^Lysm^ and wildtype mice. The DEGs with log₂FC > 1 in M2-like compared to M0-like macrophages from IL-10RαLysm mice and log₂FC > 0 in M2-like compared to M0-like macrophages from wildtype mice were further subjected to GO analysis. The enriched pathways relevant to lipid metabolism (in red) and immunoregulation were shown.

**Figure S3. TAMs in GBMs had high COX-1/2 expression**

**A** and **B**. Representative images (**A**) and statistical quantifications (**B**) of immunofluorescent analysis of pSTAT3 (green) and the pan-macrophage marker Iba1 (red) in intracranial GBMs in IL-10Rα^Lysm^ and wildtype mice. Frozen sections were immunostained with antibodies against pSTAT3 and Iba1, and counterstained with Hoechst to show nuclei. Tumors in IL-10Rα^Lysm^ mice had smaller proportions of pSTAT3^+^ macrophages relative to those in wildtype mice. Scale bar, 40 μm. (n = 5 sections for each group; mean ± s.d.; P < 0.0001; ***, P < 0.001; two-tailed unpaired student’s t-test)

**C.** UMAP visualization of cell clusters in human GBM tissues (upper) and UMAP plots showing the expression patterns of COX-1 (PTGS1) and COX-2 (PTGS2) (lower). Bar color represents gene expression level.

**D** and **E**. Heatmap showing PTGS1 (**D**) and PTGS2 (**E**) expression in different cell types in GBMs. scRNA-seq datasets from GEO database (GSE103224, GSE138794, GSE139448, GSE141385, GSE141982, and GSE148842) was analyzed and visualized using the TISCH2 online module. The horizontal axis represents the cell types, and the vertical axis stands for the scRNA-seq datasets. Highest expressions of PTGS1 and PTGS2 were observed in monocyte/macrophage populations. Color intensity represents average gene expression level calculated by log(TPM/10+1).

**Figure S4. Pharmacological inhibition of COX-1/2 suppressed pro-tumor phenotypes of macrophages**

**A.** Quantitative PCR analysis of expressions of the inflammatory cytokines TNFα and the M1-subtype macrophage markers CD80 in mouse bone marrow derived macrophages in response to cyclooxygenase inhibitors. Bone marrow cells from mouse femurs were stimulated with M-CSF for 8 days for differentiation into M0-like macrophages, followed by treatment with Loxoprofen (5 μg/ml), NS-398 (5 μg/ml), or Tolfenamic Acid (10 μM) dissolved in DMSO for 24 hours. Treatment with the cyclooxygenase inhibitors showed negligible effects on the expression of TNFα and CD80. (n = 3 biological repeats; mean ± s.d.; for TNFα, P = 0.164 for TA vs. DMSO, P = 0.236 for NS-398 vs. DMSO, P = 0.440 for LOX vs. DMSO; for CD80, P = 0.056 for TA vs. DMSO, P = 0.196 for NS-398 vs. DMSO, P = 0.10 for LOX vs. DMSO; ns, P > 0.05; two-tailed unpaired student’s t-test)

**B.** Quantitative PCR analysis of expressions of the M2-subtype macrophage markers CD206 and CD163 in mouse bone marrow derived macrophages in response to cyclooxygenase inhibitors and conditioned medium from GL261. Bone marrow cells from mouse femurs were stimulated with M-CSF for 8 days for differentiation into M0-like macrophages, followed by treatment with the COX-1/2 inhibitors for 24 hours. The cells were then cultured in GL261 conditioned medium along with the COX-1/2 inhibitors for another 24 hours. Treatment with the cyclooxygenase inhibitors showed either inhibitory or no effects on the expression of CD206 and CD163. (n = 3 biological repeats; mean ± s.d.; for CD206, P = 0.0852 for TA vs. DMSO, P = 0.0056 for NS-398 vs. DMSO, P = 0.508 for LOX vs. DMSO; for CD163, P = 0.182 for TA vs. DMSO, P = 0.103 for NS-398 vs. DMSO, P = 0.018 for LOX vs. DMSO; **, P < 0.01; *, P < 0.05; ns, P > 0.05; two-tailed unpaired student’s t-test)

**Figure S5. Treatment with the COX-1/2 inhibitor NS-398 mitigated orthotopic GBM growth**

**A**. Quantification of bioluminescent signals from representative orthotopic xenografts in mice bearing luciferase-expressing GL261-derived GBM xenografts upon NS-398 treatment. (n = 3 per group; mean ± s.d.; P = 0.666 for Day15; P = 0.020 for Day20; P = 0.038 for Day25; *, P < 0.05; ns, P > 0.05; two-tailed unpaired student’s t-test)

**B**. Statistical quantification of immunofluorescent analysis of Iba1 in intracranial GBMs treated with control or NS-398. Frozen sections were immunostained with antibodies against Iba1, and counterstained with Hoechst to show nuclei. The intensity of Iba1 signals were similar in GBMs treated with control or NS-398. (n = 5 sections for each group; mean ± s.d.; ns, P > 0.05; two-tailed unpaired student’s t-test)

**C** and **D**. Representative images (**C**) and statistical quantification (**D**) of immunofluorescent analysis of MHCII (green) and Iba1 (red) in intracranial GBMs treated with control or NS-398. Frozen sections were immunostained with antibodies against MHCII and Iba1, and counterstained with Hoechst to show nuclei. NS-398 relative to the control treatment increased the proportions of MHCII^+^ macrophages in GBMs. Scale bar, 40 μm. (n = 5 sections for each group; mean ± s.d.; P = 0.0235; *, P < 0.05; two-tailed unpaired student’s t-test)

**Figure S6. IL-10 and IL-10Rα status in microglia as part of TAMs in GBMs**

**A**. UMAP visualization of microglia and monocyte-derived (mono-derived) macrophages constituting the TAM cluster in human GBM tissues. Public 10× Genomics scRNA-Seq GEO datasets of 37742 cells from 13 cancer samples were preprocessed and integrated with R package Seurat using classical pipelines, followed by removal of batch effect with the Harmony algorithm. Unsupervised clustering was performed based on classical cell markers. The TAM cells were further re-analyzed through data normalization, dimensionality reduction, and re-clustering, followed by visualization via RunUMAP. Cellular origin of TAMs was determined by module scoring with the Seurat function AddModuleScore based on classical marker genes, including P2RY12, TMEM119, and CX3CR1 for microglia, and LST1, VCAN, S100A8, and S100A9 for mono-derived macrophages.

**B**-**E**. UMAP plots showing the IL-10 (**B** and **C**) and IL-10RA (**D** and **E**) positive cells in microglia and mono-derived macrophages. The expression data of IL-10, and I IL-10RA were extracted from the normalized expression matrix. UMAP coordinates and gene expression values for corresponding cells were obtained using Seurat's FetchData function, with positive cells defined as those exhibiting expression values > 0. Cells were visualized in UMAP space, and positive cells were highlighted to reflect the distribution patterns of the target genes across different cell subpopulations.

**F**. UMAP visualization of cell clusters in human tumor-free brain tissues (left) and UMAP plots showing the expression patterns of IL-10 (middle) and IL-10RA (right). The scRNA-seq profiles were derived from an Alzheimer’s disease-associated dataset from the publicly accessible CELLxGENE platform (<https://cellxgene.cziscience.com/>). All sequencing procedures, primary data processing, and cell-type annotations for this dataset adhered strictly to the protocols described in the original publication. Gene Expression module in the CELLxGENE platform was employed to visualize the expression distribution of the target genes. The expression levels across annotated cell populations were displayed using the platform’s integrated visualization tools, and their spatial distribution patterns were further examined in conjunction with UMAP embeddings. All expression data presented were derived from the platform's normalized expression matrices and are intended solely for qualitative assessment of transcriptional trends.

**G** and **H**. Representative images (**G**) and statistical quantifications (**H**) of immunofluorescent analysis of the microglia marker P2PY12 (green) and the pan-macrophage marker Iba1 (red) in intracranial GBMs in IL-10Rα^Lysm^ and wildtype mice. Frozen sections were immunostained with antibodies against P2PY12 and Iba1, and counterstained with Hoechst to show nuclei. Whereas few Iba1^+^ macrophages in tumor mass showed P2PY12 staining, a large proportion of P2PY12^+^ cells were detected in Iba1^+^ macrophages in the peri-tumoral benign regions, suggesting a constrained distribution of microglia in GBMs. Scale bar, 40 μm. (n = 4 sections for each group; mean ± s.d.; P = 0.0168 for wildtype; P = 0.036 for IL-10Rα^Lysm^; *, P < 0.05; two-tailed unpaired student’s t-test)
